# Perceived discrimination and health-related quality of life among Arabs and Jews in Israel: A population-based survey

**DOI:** 10.1186/1471-2458-10-282

**Published:** 2010-05-27

**Authors:** Orna Baron Epel, Giora Kaplan, Mika Moran

**Affiliations:** 1 The School of Public Health, Faculty of Social Welfare and Health Studies, University of Haifa, Israel; 2 Gertner Institute for Epidemiology and Health Policy Research, Sheba Medical Center, Tel Hashomer, Israel

## Abstract

**Background:**

Studies have shown that perceived discrimination may be associated with impaired health. The aim of this study was to assess the levels of perceived discrimination on the basis of origin and ethnicity and measure the association with health in three population groups in Israel: non-immigrant Jews, immigrants from the former Soviet Union, and Arabs.

**Methods:**

A cross sectional random telephone survey was performed in 2006 covering 1,004 Israelis aged 35-65; of these, 404 were non-immigrant Jews, 200 were immigrants from the former Soviet Union and 400 were Arabs, the final number for regression analysis was 952. Respondents were asked about their perceived experiences with discrimination in seven different areas. Quality of life, both physical and mental were measured by the Short Form 12.

**Results:**

Perceived discrimination on the basis of origin was highest among immigrants. About 30% of immigrants and 20% of Arabs reported feeling discriminated against in areas such as education and employment. After adjusting for socioeconomic variables, discrimination was associated with poor physical health among non-immigrant Jews (OR = 0.42, CI = 0.19, 0.91) and immigrants (OR = 0.51, CI = 0.27, 0.94), but not among Arabs. Poor mental health was significantly associated with discrimination only among non-immigrant Jews (OR = 0.42, CI = 0.18, 0.96).

**Conclusions:**

Perceived discrimination seemed high in both minority populations in Israel (Arabs and immigrants) and needs to be addressed as such. However, discrimination was associated with physical health only among Jews (non-immigrants and immigrants), and not among Arabs. These results may be due to measurement artifacts or may be a true phenomenon, further research is needed to ascertain the results.

## Background

Discrimination may be based on race/ethnicity, origin, religion, culture, social-class, age and gender: people are distinguished and treated unfavorably by others due to their belonging to a specific group [1]. Discrimination can express itself at the institutional, structural or interpersonal level, depending on politics, policies, and norms of behavior in a specific society [1].

Long-term perceived discrimination can lead to the accumulation of stressors over the life course [2]. Such prolonged stress may exert an effect on health [3-6].

Studies consistently report the link between perceived discrimination and mental health, namely more individual experiences of discrimination are associated with poor mental health and mental diseases [4,7-9]. Discrimination was also reported to be associated with many physical health measures, including high blood pressure [10,11], respiratory problems [12], self-rated health [13,14] and chronic health conditions [15,16]. Mental health may be affected by perceived discrimination more than physical health [17].

Most information about discrimination and health has come from studies performed in the USA among Black Americans, Hispanic Americans [18] and Asian-Americans [6,8]. Studies from other countries have looked at immigrants and ethnic minorities in Western countries, such as Canada [19,20], England [12], Ireland [21], the Netherlands [9], Denmark [22], Finland [23] and New Zealand [24]. These studies also found that people reporting perceived discrimination have poor mental as well as physical health. To better understand the effect of discrimination on health, studies should look at discrimination in other social contexts. Israel may serve as such a society, where indigenous and immigrant minorities with different cultural backgrounds co exist side by side with the majority, and have similar rights to social and health services [25]. The historic development formed a country with three major population groups: Jews born in Israel or residing in Israel most of their life, immigrants, and Arabs. The population in Israel at the end of 2006 numbered 5.4 million Jews and 1.4 million Arabs. The immigrant population includes the current immigration groups arriving in Israel since 1990. From 1990 to 2006 a large immigration wave numbering 937,100 immigrants (13.3% of the population in 2006) arrived in Israel from the former Soviet Union (fSU). About 55% of them entered the country during the first five years of the immigration wave and about 14% of them entered from 2000 on [26]. Under the Law of Return, all Jews can immigrate to Israel regardless of their health status, and on arrival they are entitled to all national welfare and healthcare services. Studies have reported worse self-reported health among these immigrants [27] as well as high prevalence rates of self-reported chronic disease [28]. Similar levels of use of healthcare services by non-immigrant Jews and immigrants have also been reported [29]. Non-immigrant Jews and fSU immigrants differ in background, culture and language, but they are not segregated in their living areas.

Arabs living in Israel comprised 19.8% of the population in 2006. Arabs and Jews differ in ethnicity, religion, culture, and language. Arabs are largely an underprivileged minority with a history of disadvantage in income, education and employment and are a more collective society even though they are regarded as a society in transition [30,31]. The Arabs are mostly segregated in their living areas, and less than ten percent live in mixed towns or cities; most Arab communities are rural. Despite enjoying full citizenship status, the Arab minority is subject to various forms of discrimination that may contribute to social and economic disparities between them and the Jewish majority. It has been suggested that discrimination does play a part in the income disparities between Arabs and Jews [32,33].

The mortality and morbidity of the Arab population is higher than in the Jewish population [34-36]. As Israel has a comprehensive National Health Insurance Law, disparities due to access to healthcare services are low and Arabs' use of healthcare services is similar to that reported by Jews [37].

Israel's diverse society, with its national healthcare service, may be an interesting setting to study the effects of discrimination on health and we hypothesis that we will find higher levels of discrimination among Arabs and immigrants compared to non-immigrant Jews and that discrimination will be associated with health in all three groups.

The aims of this study were (1) to assess perceived levels of discrimination on the basis of origin and ethnicity, among three population groups in Israel: non-immigrant Jews, immigrants from the fSU, and Arabs; (2) to measure the association of perceived discrimination with mental and physical health in each population group.

## Methods

### The sample

This cross-sectional study was based on a random sample of the Israeli population aged 35-65 years in 2006. Two random samples of telephone numbers were drawn from a computerized list of subscribers to the national telephone company: one for Arab subscribers including Arab towns and villages, and one for members of the Jewish majority. The Arab sample does not include Arabs living in mixed cities such as Jerusalem and Haifa as it is not possible to sample them separately. The immigrant respondents were sampled from the Jewish list by self-reporting of year of immigration and country of origin. In 2007, telephone lines (not cellular telephones) were present in 90% of Jewish households and in 65% of Arab households, this may under-represent the poorer Arabs in the survey [38,39]. Excluded were fax numbers, disconnected numbers, commercial numbers, and households where there was no reply (after six attempts on different days) or no available resident of the target age. This left 1,541 eligible households in the sample. Immigrants were over-sampled until a quota of 200 interviews was reached. Immigrants not from the fSU were not included in the study. A total of 1,004 respondents, men and women, completed the questionnaire, yielding a response rate of 60% among Jews and 74% among Arabs. The final database was 404 non-immigrant Jews, 200 immigrants and 400 Arabs. The survey was conducted between January and February 2006 at the Haifa University Survey Center.

Due to missing data on some questionnaires the sample analyzed in the final regressions consisted of 952 completed questionnaires.

### The questionnaire

The questionnaire had several parts and covered a wide range of socioeconomic and demographic variables to measure perceived discrimination and health status. The Hebrew questionnaire was translated into Arabic and Russian, then back-translated into Hebrew to ensure accuracy. Arab professionals speaking both Arabic and Hebrew, and familiar with Israeli-Arab culture, validated the Arabic translation from the Hebrew questionnaire, and confirmed that the questions had the same meaning as in Hebrew; the Russian questionnaire underwent the same process. Much thought was put into the exact wording to decrease differential understanding of the questions in the three groups. A pretest with 15 people from each population group (45 in all) to ensure culture adaptation of the questionnaire encountered no obstacles. The questionnaire was administered over the telephone by trained interviewers from the relevant population group for each language: Hebrew, Arabic, and Russian.

No official ethical approval was sought for this study. At the time of the research official ethical approval was not needed in Israel for this kind of study which was a random digit dial survey (no data from lists of patients or clients were used) and no medical information was obtained from other sources. Even so, the highest ethical standards were adhered to and maintained in the study's procedures and methods. The following steps were followed by the interviewers: they introduced themselves; they briefly described the survey topic; they identified the person and organization conducting the research; described the purpose of the research and gave a "good faith" estimate of the time required to complete the interview; they also promised anonymity and confidentiality; the interviewers mentioned to the participant that participation is voluntary and that item-nonresponse is acceptable. Finally, permission to begin was asked. Informed consent was considered to have been obtained when potential participants agreed to answer the questionnaire.

### Variables

All variables were self-reported. Arabs were defined as those describing themselves as Arab Muslims, Druze, or Arab Christians. Immigrants were those who reported arriving in Israel since 1989 from the fSU. Most of the immigrants (75%) arrived in Israel between 1989 and 1996, and 8% of them arrived after 2000. Non-immigrant Jews were those living in Israel before 1989. To measure subjective socioeconomic status (SSS) participants were invited to think of a ladder with ten rungs as representing where people stand in Israeli society. The interviewee gauged his or her SSS on the ladder on a scale from 1 to 10 [40,41]. Employment status was categorized as working (1) or not working (0) (unemployed, retired, housewife). Education was assessed by the highest degree the respondent attained, and three categories were formed: not completing high school (1), non-academic studies including high school or any other studies beyond high school that did not furnish an academic degree (2), or having a degree from a university or college (3). Men were categorized 1 and women 2.

The "Short Form 12" (SF12) questionnaire, validated in Hebrew [42], served to measure quality of life as related to physical and mental health [43]. Six items measured mental health (one item on vitality, one on social functioning, and two each on role-emotional and mental health). Another six items measured physical health (one on bodily pain, one on general health, and two each on physical functioning and role-physical). Scores were transformed to a scale of 100, where 100 was optimal health and 0 was poor health; a mean score was calculated for each individual. Since physical and mental health variables were not normally distributed, they were dichotomized around the median. Both mental and physical health status were categorized as suboptimal (0), with scores from 0 to 79.99, and optimal (1), with scores of 80 and above, so that about half the respondents were classified as having suboptimal health and the other half as having optimal health.

The perceived discrimination questionnaire was adapted from the instrument developed and validated by Krieger and colleagues [17]. Respondents were asked to assess the frequency they felt discriminated against, or unfairly treated, because of their origin or ethnicity, in seven settings: education, finding a job, the workplace, obtaining housing, receiving healthcare, dealing with public institutions, and in public places (additional file 1). The Hebrew word chosen to render the word *ethnicity *in the English version - which can be translated into English as "origin" - has a much broader meaning than ethnicity and as such is more appropriate for the immigrant population. This concept was also used in the Arabic and Russian questionnaires. For each of the seven settings listed above, the respondent was asked to assess the frequency of experiencing discrimination. A choice of four levels was offered, from 1-"not at all" to 4-"frequently". A mean score was calculated including the settings in which discrimination took place. The continuous measure was used in the correlation and regression analysis. In addition, the level of feeling discrimination for each setting was dichotomized into two groups: never ("not at all") (0) and at least sometimes ("infrequently", "sometimes", and "frequently" together) (1). An overall dichotomized score was calculated including all settings, where 0 represents respondents who reported no experience of discrimination in any setting, and 1 represents respondents who experienced at least some discrimination in at least one setting. The dichotomized variable was used for the descriptive analysis shown in table 2.

### Statistical analysis

Chi-square analysis was used to identify differences in the socioeconomic variables and in discrimination between the three population groups. Spearman's correlations were applied to assess crude associations between discrimination and health. Multivariable logistic regression analysis was performed for each group separately, with physical and mental health as the dependent variables, to assess the association between health and discrimination, after adjustment for other variables associated with health. The odds ratios (OR), 95% confidence intervals (CI) and p-values are presented in the tables. Variables found to be associated with health in the crude analysis were entered into the models. Age, SSS, and discrimination were entered into the model as continuous variables. Another multivariable regression model for the entire population was run to assess whether Jews and Arabs differed significantly in the association between discrimination and health; this was measured by the interactions of discrimination and population group with health.

Statistical significance was set at a p value of less than 0.05. SPSS version 14.0 was used for the analysis.

## Results

### Socio-demographic characteristics

The study population consisted of three groups, non-immigrant Jews (404), immigrants from the fSU (200) and Arabs (400). Of the Arabs, 71% were Muslim, the rest Christian or Druze. Socioeconomic measures are presented in table 1.

**Table 1:** Demographic, socioeconomic and health characteristics by population group [percent and (number), mean and (standard deviation)]

Characteristics		Non-immigrant Jews	Immigrants	Arabs
**Total**		404	200	400
**Gender**	**Men**	40.3 (163)	40.0 (80)	42.5 (170)
	**Women**	59.7 (241)	60.0 (120)	57.5 (230)
Age* (years)	**Mean (SD)**	49.1 (8.8)	50.2 (8.7)	45.0 (8.1)
Education*	**Less than high school**	22.2 (89)	3.5 (7)	58.4 (233)
	**High school or more**	32.2 (129)	41.0 (82)	24.1 (96)
	**Academic** **degree**	45.6 (183)	55.5 (111)	17.5 (70)
Employment*	**Yes**	77.7 (313)	78.3 (155)	49.3 (197)
	**No**	22.3 (90)	21.7 (43)	50.8 (203)
Subjective Socioeconomic Status*	**Mean (SD)** **(range 1-10)**	6.43 (1.8)	4.22 (1.6)	5.63 (2.3)
Physical health related quality of life*	**Mean (SD)** **(range 1-100)**	82.3 (20.9)	72.3 (24.4)	71.5 (29.0)
Mental health related quality of life*	**Mean (SD)** **(range 1-100)**	77.8 (20.5)	66.8 (24.6)	68.2 (25.6)

*differences between population groups p < 0.0001

These characteristics reflect the expected differences between the population groups in Israel, where Arabs and immigrants have a lower socioeconomic status than non-immigrant Jews (excluding education among immigrants). Arabs were significantly younger and less educated, and with relatively fewer of them in the work force; immigrants were older and more educated, and their SSS was lower than that of either non-immigrant Jews or Arabs.

### Discrimination

The respondents were asked about their perceived experiences with discrimination based on their origin or ethnicity in several life areas, and the findings are presented in table 2.

The group with the largest percent of respondents reporting perceived discrimination in at least one of the settings measured were immigrants (71.5%). Of the Arabs, 40.5% reported at least some experience with discrimination, and of non-immigrant Jews 21.0% reported this. Generally, perceived discrimination was high in areas such as employment, the education system, public places, and public institutions. The least reported settings for discrimination were in obtaining housing and using the healthcare system. Among Arabs, the education system was the most frequently cited as a setting for discrimination, whereas among immigrants discrimination in public places was most frequently cited.

**Table 2:** Levels of perceived discrimination by population group (number, percent and p*-value).

Area of discrimination		Non-immigrant Jews		Immigrants		Arabs	
		
		N	%	N	%	N	%
**Education system**	None	359	89.8	132	68.4	312	80.0
	Some**	41	10.2	61	31.6	78	20.0
	P	<0.0001					
**Finding employment**	None	368	91.3	129	66.2	308	80.8
	Some**	35	8.7	66	33.8	73	19.2
	P	<0.0001					
**At the work place**	None	368	91.1	125	63.2	314	83.7
	Some**	36	8.9	73	36.9	61	16.3
	P	<0.0001					
**Obtaining housing**	None	393	99.0	179	90.9	341	92.9
	Some**	4	1.00	18	9.1	26	7.1
	P	<0.0001					
**The health care system**	None	393	97.3	175	88.8	370	92.7
	Some**	11	2.7	22	11.2	29	7.3
	P	<0.0001					
**At public institutions**	None	384	95.0	130	66.4	310	80.6
	Some**	20	5.0	66	33.7	75	19.5
	P	<0.0001					
**Public places**	None	371	92.1	122	61.0	320	81.0
	Some**	32	7.9	78	39.0	75	19.0
	P	<0.0001					
Total***	**None**	319	79.0	57	28.5	238	59.5
	Some**	85	21.0	143	71.5	162	40.5
	**p**	<0.0001					

* chi square analysis

**includes at least some perceived discrimination

*** summary of all areas of discrimination

### Discrimination and health

There was a significant negative correlation between discrimination due to origin or ethnicity and both mental and physical health. Spearman's correlation coefficient for physical health and discrimination was -0.14 (p < 0.0001), and for mental health and discrimination -0.21 (p < 0.0001) (data not presented). The higher the perceived discrimination, the lower were both mental and physical health. These associations were significant among both non-immigrant Jews and immigrants; among Arabs the associations were lower and not significant.

Logistic-regression models were used to adjust for age, gender, employment, education, and SSS, as these variables are known to be associated with health. The analysis was run separately for each population group because the interactions between discrimination and population group for the entire population were significant in a logistic regression model (p = 0.004).

After adjustment for these variables in a logistic-regression model run for each population group separately (table 3), perceived discrimination was still independently and significantly associated with physical health in non-immigrant Jews (OR = 0.42, CI = 0.19, 0.91) and immigrants (OR = 0.51, CI = 0.27, 0.94). Among Arabs, OR was above 1 but not statistically significant (OR = 1.48, CI = 0.93, 2.34). As the interaction between population group and discrimination was statistically significant, an association between discrimination and physical health seems to exist among Jews but not among Arabs (data not presented).

**Table 3:** Variables associated with physical health related quality of life in logistic regression models, by population group.

Independent variables in the model	Non-immigrant Jews N = 383			Immigrants N = 187			Arabs N = 382		
	
	OR	CI	p	OR	CI	p	OR	CI	p
**Discrimination**	**0.42**	0.19, 0.91	0.03	**0.51**	0.27, 0.94	0.03	1.48	0.93, 2.34	0.10
**Age**	0.98	0.96, 1.01	0.29	0.98	0.94, 1.02	0.27	**0.97**	0.94, 0.998	0.04
**Gender**	0.73	0.45, 1.21	0.22	**0.26**	0.13, 0.53	< 0.0001	0.62	0.37, 1.04	0.07
**Employment status**	1.45	0.82, 2.57	0.20	**2.99**	1.20, 7.47	0.02	**1.70**	1.01, 2.88	0.047
**Education**	**1.68**	1.23, 2,30	0.001	0.93	0.50, 1.75	0.83	**1.66**	1.19, 2.33	0.003
SSS**	**1.19**	1.04, 1.37	0.01	1.25	0.98, 1.57	0.07	**1.16**	1.05, 1.29	0.005

[Odds Ratio* (OR), 95% Confidence Interval (CI) and p value]

* ORs adjusted for all variables in the model, health was categorized as: 0 for suboptimal health and 1 for optimal health

** Subjective Socioeconomic Status

Poor mental health was associated with perceived discrimination only among non-immigrant Jews (OR = 0.42, CI = 0.18, 0.96), but not among immigrants and Arabs, after adjustment for socioeconomic variables, gender, and age (table 4).

**Table 4:** Variables associated with mental health related quality of life in logistic regression models by population group.

Independent variables in the model	Non-immigrant Jews N = 383			Immigrants N = 187			Arabs N = 382		
	
	OR	CI	p	OR	CI	p	OR	CI	p
**Discrimination**	**0.42**	0.18, 0.96	0.04	0.75	0.41, 1.37	0.35	0.68	0.48, 1.08	0.11
**Age**	1.02	0.99, 1.05	0.17	1.01	0.97, 1.05	0.57	0.996	0.97, 1.02	0.76
**Gender**	**0.48**	0.29, 0.77	0.002	0.59	0.31, 1.13	0.11	1.03	0.62, 1.71	0.91
**Employment status**	0.91	0.51, 1.63	0.76	1.96	0.76, 5.04	0.17	**1.95**	1.15, 3.30	0.01
**Education**	1.29	0.95, 1.75	0.10	1.06	0.59, 1.92	0.84	1.04	0.76, 1.42	0.81
SSS**	**1.45**	1.25, 1.67	< 0.0001	**1.44**	1.14, 1.82	0.002	**1.25**	1.12, 1.39	< 0.0001

[Odds Ratio* (OR), 95% Confidence Interval (CI) and p value]

* ORs adjusted for all variables in the models, health was categorized as: 0 for suboptimal health and 1 for optimal health

** Subjective Socioeconomic Status

## Discussion

Perceived discrimination was reported more frequently in the minority groups (Arabs and immigrants) than in the majority group (non-immigrant Jews) as hypothesized. Many studies on immigrants in other countries found that their perceived discrimination was greater than that of the majority population [6,22,24,44]. In addition, many of those reporting perceived discrimination reported poor physical and mental health. This is in keeping with other studies conducted in the last 15 years [1,4-6,24]. However, the association between physical health and discrimination was observed only among Jews (non-immigrants and immigrants) after adjusting for socioeconomic status. Among Arabs discrimination seems unassociated with physical health contradicting our hypothesis. Mental health was associated with discrimination only among non-immigrant Jews.

This is the first study to analyze quantitatively the association between health and discrimination in Israel where two very different minorities live, an immigrant population living within the Jewish non-immigrant population and an indigenous Arab population that is segregated to some extent from the Jewish majority.

The study has some limitations. The data are cross sectional; hence provide no basis for causal directionality. Discrimination may cause poor health; however, poor health may also increase levels of perceived discrimination. Yet no evidence of this has been found [45]. Another major limitation may be differential reporting of discrimination among Arabs, immigrants and non-immigrant Jews, each group may comprehend the questions differently, and the willingness of each group to report discrimination may vary for different reasons. Response bias may vary in the three groups: Arabs may under-report discrimination, and immigrants may over-report it. Arabs may under-report discrimination due to unwillingness or fear of expressing their real feelings to a stranger on the telephone. Over reporting by immigrants may be due to their expectations of being received by the Israeli society in a more positive way. In addition, self-selection caused by non-respondents may differ between the three groups. Among Arabs, the lower socioeconomic class may be under-represented due to lower rates of families with telephone lines among the poor or lower rates of response to the survey among the poor. This too may decrease levels of reported discrimination if discrimination is higher among the poor. Moreover, since health status is socio-economic related it is possible that non-respondents were sicker and with more feelings of discrimination and may explain to some extent why we found no association between perceived discrimination and physical and mental health among Arabs. However, the association between discrimination and health on the various socioeconomic levels do not seem to differ, so no bias would be expected.

Perceived discrimination in the three groups arises in different social, historical and political contexts. Discrimination may be perceived by immigrants partly due to a sense of cultural estrangement and the non-realization of the immigrant's high expectations for a better life in Israel. Furthermore, Israeli society may actually discriminate against the immigrants, justifying their perceptions. Within one generation it is expected that this group and their descendents will report similar perceived discrimination as the non-immigrants report. It is expected that their acculturation will eliminate the differences between them and other non-immigrant Jews; the immigrants will be indistinguishable from the non-immigrants. Discrimination among the non-immigrant Jews may be attributed to the heterogeneity of the non-immigrant Jewish society that includes Jews from different origins such as the Sephardic Jews and the Ashkenazi Jews, some of these Jews may still have feeling of discrimination on the basis of their origin.

The perceived discrimination of the Arab community may have a more profound basis including institutional discrimination and prejudice against Arabs, where less funding is available for the Arab communities and discrimination in attaining jobs is frequent, in addition to prejudice on behalf of the Jewish population [30,31,33].

For both immigrants and Arabs the healthcare services seem to be a system with comparatively little perceived discrimination. This may be because some of the employees of the healthcare services are Arabs and immigrants, and they work with their respective communities. Housing discrimination was not reported as a major area of discrimination, as Arabs usually do not try to buy or rent housing in the Jewish community. This does not mean there is no housing discrimination towards Arabs; on the contrary, it is institutionalized discrimination and may contribute to the continuing residential segregation of the Arab population [46]. The discrimination measure used in this study was developed for studying discrimination in the USA [17] and did not include other areas of discrimination that may be unique to Israel, such as that fact that most Arabs do not serve in the military and discrimination in national politics, further studies should look at these aspects too.

A major difference between Arabs and immigrants is their relationship with the majority Jewish population. The immigrants live among the majority of non-immigrant Jews and expect to be a part of the Jewish society, whereas the Arabs do not; they perceive themselves as Arabs within a Jewish society. These differences may affect both the levels of perceived discrimination and the association between health and discrimination. The finding that among Arabs perceived discrimination was not associated with health can be explained by the Arabs' residential segregation. Lower levels of contact with the majority group may prevent ongoing perceived discrimination, including exposure to everyday hassles and elevated stress, among the Arabs compared to Jews. Therefore, the Arabs' health could be less affected. Another explanation may be that the Arabs do not expect to be part of Israeli Jewish society and their ethnic identity serves as a coping mechanism preventing prolonged stress due to discrimination; this may obviate ill effects on health. A similar condition was found in African Americans, where racial identity was a protective factor in buffering the negative impact of discrimination [47].

Efforts are necessary to lower the level of actual and perceived discrimination in both the Arab population and among immigrants, as Israel prides itself on being a nation founded on democracy and equality. The chances of this happening may increase mainly after the relationship of Israel and its neighbors improves. At present a reduction in institutional discrimination (especially as regards the Arab population) and more education for tolerance and open-mindedness could bring about a positive change.

There is a need to repeat this finding with other measures of health so as to ascertain that the results are not an artifact of measurement. If this phenomenon is not an artifact further research is needed on the impact of segregation in living areas and perceived identity as possible explanations for the differences in the association between discrimination and health in different population groups.

## Conclusions

Individuals that report perceived discrimination also report poor health, however, this is observed only in the Jewish population but not in the Arab population. Further research on the association between perceived discrimination and health should be performed firstly to ascertain this is a true phenomenon and not an artifact. If this is a true phenomenon further factors serving as coping mechanisms that prevent the ill effect of perceived discrimination in specific communities should be investigated.

## List of abbreviations

fSU: former Soviet Union; SSS: Subjective socioeconomic status.

## Competing interests

The authors declare that they have no competing interests.

## Authors' contributions

OBE initiated the study, performed the analysis and wrote the manuscript, GK initiated the study, and helped write the manuscript, MM helped with the collection and the analysis of the data. All authors read and approved the final manuscript.

## Pre-publication history

The pre-publication history for this paper can be accessed here:

http://www.biomedcentral.com/1471-2458/10/282/prepub

## Supplementary Material

Additional file 1 The discrimination questionnaire used in this study. Click here for file
