# Supplementary material for: Perceived discrimination and health-related quality of life among Arabs and Jews in Israel: A population-based survey
Source: BMC Public Health. 2010 May 27;10:282. doi: 10.1186/1471-2458-10-282 (PMC2891650; doi:10.1186/1471-2458-10-282)
Supplement: Additional file 1 — The discrimination questionnaire used in this study. [file 1471-2458-10-282-S1.DOC]

**Discrimination questionnaire**

Orna Baron-Epel, Giora Kaplan and Mika Moran

Based on a measure by Krieger and colleagues

Krieger N, Smith K, Naishadham D, Hartman C, Barbeau EM: **Experiences of discrimination: Validity and reliability of a self-report measure for population health research on racism and health**. *Social Science & Medicine* 2005, **61**(7):1576-159.

This is a translation from the Hebrew questionnaire we used.

I will read to you a number of situations. In each of these situations at what frequencies have you felt discriminated against because of your ethnic background or origin:

|  | **Frequently** | **Sometimes** | **Infrequently** | **Never** |
| --- | --- | --- | --- | --- |
| 1. **At school** | 4 | 3 | 2 | 1 |
| 1. **Getting a job** | 4 | 3 | 2 | 1 |
| 1. **At work** | 4 | 3 | 2 | 1 |
| 1. **Renting or buying a place to live** | 4 | 3 | 2 | 1 |
| 1. **Getting health care** | 4 | 3 | 2 | 1 |
| 1. **When turning to public institutes** | 4 | 3 | 2 | 1 |
| 1. **In the street or in public places** | 4 | 3 | 2 | 1 |
